# Financial impact of nursing professionals staff required in an Intensive Care Unit^1^

**DOI:** 10.1590/1518-8345.1274.2818

**Published:** 2016-11-21

**Authors:** Thamiris Ricci de Araújo, Mayra Gonçalves Menegueti, Maria Auxiliadora-Martins, Valéria Castilho, Lucieli Dias Pedreschi Chaves, Ana Maria Laus

**Affiliations:** 2 MSc, RN, Hospital das Clínicas, Faculdade de Medicina de Ribeirão Preto, Ribeirão Preto, SP, Brazil.; 3 PhD, Professor, Faculdade de Medicina de Ribeirão Preto, Ribeirão Preto, SP, Brazil.; 4 PhD, Associate Professor, Escola de Enfermagem, São Paulo, Universidade de São Paulo, São Paulo, SP, Brazil.; 5 PhD, Associate Professor, Escola de Enfermagem de Ribeirão Preto, Universidade de São Paulo, PAHO/WHO Collaborating Centre for Nursing Research Development, Ribeirão Preto, SP, Brazil.

**Keywords:** Intensive Care Units, Nursing, Workload, Hospital Costs, Costs and Cost Analysis

## Abstract

**Objective:**

to calculate the cost of the average time of nursing care spent and required by patients in the Intensive Care Unit (ICU) and the financial expense for the right dimension of staff of nursing professionals.

**Method:**

a descriptive, quantitative research, using the case study method, developed in adult ICU patients. We used the workload index - Nursing Activities Score; the average care time spent and required and the amount of professionals required were calculated using equations and from these data, and from the salary composition of professionals and contractual monthly time values, calculated the cost of direct labor of nursing.

**Results:**

the monthly cost of the average quantity of available professionals was US$ 35,763.12, corresponding to 29.6 professionals, and the required staff for 24 hours of care is 42.2 nurses, with a monthly cost of US$ 50,995.44.

**Conclusion:**

the numerical gap of nursing professionals was 30% and the monthly financial expense for adaptation of the structure is US$ 15,232.32, which corresponds to an increase of 42.59% in the amounts currently paid by the institution.

## Introduction

In Intensive Care Units (ICU), the activities carried out by different professionals represent the most significant component in cost accounting, and the nursing staff have represented 30 to 35% of total costs^1^_._

The urgency in cost control imposes on health institutions the need for a careful analysis of the nursing professionals staff that is needed, in view of the fact that it represents a significant financial burden in critical units^2^.

This aspect has required of nurses the knowledge of different methods of providing staff to the ICUs, so as to obtain success in the negotiations with hospital administrators in hiring human resources for this area^3^, in order not to experience quantitative restrictions for these professionals. However, it is necessary to associate the staffing design with methods that provide the identification of the cost of nursing care.

In Brazilian health institutions, to evaluate the costs of nursing staff, it is used several global analysis methodologies, but knowledge of the characteristics of the patients becomes essential, in order to allow these methods to grasp specificities of care and their demands in terms of working time. With this set of information, researchers can estimate the cost and justify the need for investment to be made^4^. 

The instruments to quantify the work of nurses have been used to obtain the costs of care, they are capable of discriminating the nursing participation in the total cost of the unit^1^. In this context, the *Nursing Activities Score* (NAS) has been found as a reliable and valid instrument to measure ICU workload^5^_._ It can be used as a management tool, cost planning, audit in ICU, and also in estimates of monetary values involved in nursing care provided to critically ill patients^6^. This is an index that allows the budget calculation of nursing service from actual data of care hours needs required by patients^1^.

Given the strategic position of the costs in health institutions, research is necessary to enable the assessment of the performance of the units in order to provide subsidies for the planning, control and decision making of the professional staff in the ICU.

This study aimed to calculate the cost of the average nursing care time spent and required by patients admitted to the Intensive Care Unit (ICU) and the financial expense for the adequacy of the nursing professional staff.

## Method

This is a descriptive study, quantitative approach in the case study method, developed in an adult patients ICU in a large and highly complex teaching hospital in the state of São Paulo, Brazil.

The adult intensive care unit has 14 hospital beds, of which nine are intended for clinical and surgical patients and five beds for patients of Cardiology. The study population was comprised of ICU patients, over 18 years, regardless of sex, diagnosis, length of stay in the unit or type of treatment.

Data collection was performed by the researcher using a methodology^7^ ^)^ consisting of the following steps:

### Identification of nursing staff workload

To this end, the prospective application of NAS index was applied, which was constituted on direct observation and evaluation of patients admitted to the unit. Additional information regarding the events of the last 24 hours was obtained from medical records.

The use of prospective NAS provides more reliable results when measuring the nursing workload in ICUs. This application form is intended to assist the patient in full and according to their needs, free of interference related to the organizational structure of the institution that can hamper the practice of right kind of care^8^.

The NAS is divided into seven major categories, a total of 23 items, with weights ranging from 1.2 to 32.0 and comprises 80.8% of nursing activities. The assessed care categories are: basic activities (monitoring and controls, laboratory investigations, medication, hygiene procedures, care of drains, mobilization and positioning, support and care to families and patients, and administrative and managerial tasks); ventilatory support; cardiovascular support; renal support; neurological support, metabolic support and specific interventions. The total score can reach a maximum of 176.8%, which represents the percentage of time spent by nurses per shift, in direct patient care^6^.

For the conformation of the sample, its application was performed once a day for a period of thirty days in the months of March and April 2014, resulting in a sample that reflects the profile of nursing interventions needs required by patients in this ICU.

### Identification of the average time of care dispensed to patients by professional category

The average time was obtained by the average amount of each professional category that was working in the unit during the sample period, the average daily number of patients and the working day^7^: 



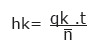



 In which:

*h_k_* = average time of care (hours), per patient, according with the professional category *k;*

*q_k_* = daily average number of professionals of the professional category *k*;

*t* = working hours of professionals,


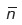
 = average daily number of patients.

### Identification of the average time of care required by patients 

The daily application of NAS allowed the sum of the values of the set of patients, yielding the total daily NAS. In a second step, this value was divided by the number of hospitalized patients in the day, obtaining thus the average daily NAS of the period investigated, applied to a new equation^7^: 



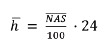



In which:


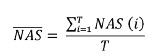
= NAS average value of a sample of T patients;


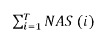
= sum of NAS of each patient i, from i = 1 to i = T;

T = number of patients shown in the period;

24/100 = corresponding relationship to 24 hours per 100 NAS points.

### Daily quantity of nursing professionals required

Through the sum of the NAS values of each patient, the NAS daily total was obtained. Then we proceeded to the sum of all NAS total daily values and divided by the number of sampled days, obtaining a mean value, which is the unit's workload used in the equation below^7^, as follows:



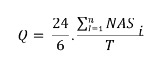



In which:


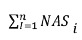
= NAS sum of each patient

### Gauging the cost of Direct Labor (DL)

The cost of the Direct Labor represents the expenditure on personnel working in the production of the products or services. It includes salaries, social charges arising from the labor and social security law, and all other expenses that are related to workers^9^. 

To obtain the cost of direct labor, the monthly pay composition and the professional category were used, based on the nursing staff of professionals working in the ICU, in the data collection period.

Data on monthly wage composition were provided by the Human Resources Department of the institution comprised of the average wage of the category; hazardous job pay; Executive gratification; Incentive; holiday aliquot, namely 1/12 to 1/3 of the monthly salary composition; social charges: Pension Plan and Service Time Guarantee Fund (FGTS), part of the 13th salary, namely, 1/12 the average wage category. It was decided, in this study, the use of baseline salary of different professional categories in the institution.

The cost of direct labor was calculated by dividing the monthly salary composition of each professional category and monthly working time considered in this study, 111 hours, which corresponds to the month of March 2014. 

The currency used for the different stages was the US currency (dollar), considering their average month quotation on March 2014 of USD 2.33^10^. For calculation purposes, we considered the values of daytime hours.

To obtain the financial value of the average time spent per patient care in each work shift and occupational category, we used an equation that converts the average quantity of available professionals in morning, afternoon and night shifts, in hours of care^7^_._

For the hourly cost of care required by patients, the percentages of time spent on each work shift were projected, achieved through the application of a direct simple cross-multiplication.

This logical sequence made possible to know the approximate cost of assistance expended and required by patients in different shifts of 24-hour care, and the monetary difference necessary to adequate the staff of the unit under study.

To know the variations that occurred in the subtotal and total costs of assistance expended and required, we used the change in costs calculation, which includes the relationship between a previous value and a further value subtracting 1 and multiplying by 100^11^.

The data were sorted and stored in spreadsheets developed in Microsoft Excel 2010 program and subsequently analyzed using descriptive statistics.

Regarding ethical aspects, the patients who presented conditions to decide for their participation in the study signed the Informed Consent form (ICF) and in the impossibility of their decision, those responsible for them were contacted and consulted and by signing the Informed Consent Form, ensured compliance with the provisions of the Resolution 466/12 of the National Health Council. 

The research is approved at the Research Ethics Committee of the institutions involved, under the protocol number CAAE 24373213.5.0000.5393.

## Results

77 patients were included, with a predominance of males (n=44; 57%), mean age 57.3 years (SD=15.9), mean length of stay of 7.3 days (SD=7.7), coming from the inpatient units (n=29; 38%), with clinical conditions (n=62; 80.6%) and causes of hospitalization for cardiovascular diseases (n=27; 35%). The outcome obtained in 79% of cases was the discharge from the unit. 

The NAS instrument was applied 369 times and the average daily score was 85.6 (SD = 4.3). The actual average number of nursing staff was 8.4 nurses and 21.2 technicians to meet the average quantity of 12.3 patient-days.

The average time spent in care amounted to 14.4 hours/day/patient (100%), broken down in 4.1 hours (28.5%) by nurses and 10.3 (71.5%) by technicians.

The average daily NAS score is equivalent to 20.5 hours per patient nursing care. From those hours, 5.8 (28.5%) should be dispensed by nurses and 14.7 (71.5%) by nursing staff. Thus, the amount required professionals for 24 hours of care is 42.2 professionals, of which 12 (28.5%) should be nurses and 30.2 (71.5%) technicians.

Regarding the cost of Direct Labor, Table 1 shows the different elements of the monthly salary composition of the unit of nursing professionals investigated.

**Table 1:** Calculation of monthly salary composition of nursing professionals in the Intensive Care Unit (ICU). Ribeirão Preto, SP, Brazil, 2014

Monthly Salary Composition Nursing	Nurses US$*	Technicians US$*
a. Average salary of the category	250,74	165,98
b. Hazardous job pay	62,15	62,15
c. Executive bonus	377,94	275,54
d. Incentive	394,85	313,73
e. Vacation aliquot (1/12 of 1/3 of a+b+c+d)	30,16	22,70
f. Social charges		
Pension plan (22,53% of a+b+c+d)	244,61	184,16
FGTS (Pension Plan and Service Time Guarantee Fund) (8% of a+b+c+d)	86,85	67,11
g. Part of the 13th salary (1/12 Average salary of the category)	20,90	13,83
Total	1.468,20	1.105,20

*Quotation from March 2014 US$ 1,00 = R$ 2,33

The unit value of professional work time according to the nursing category was US $ 13.23 / hour for nurses (US $ 1,468.20 / 111 hours) and US $ 9.96 / hour for nursing technicians (US $ 1105.20 / 111 hours). 

Tables 2 and ^3^show the cost of time spent and care required by professional category and the different shifts.

**Table 2:**
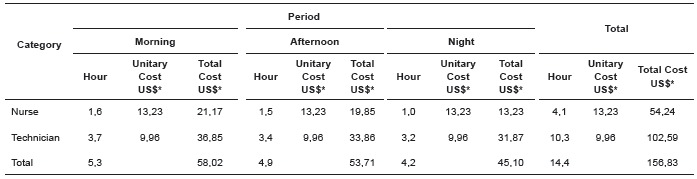
Cost of the assistance dispensed hour per patient within 24 hours, according to the professional category and the work shift. Ribeirão Preto, SP, Brazil, 2014

*Quotation from March 2014 de US$ 1,00 = R$ 2,33

**Table 3:**
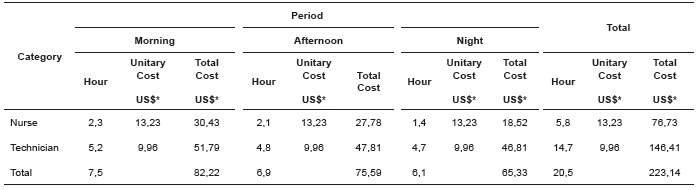
Cost of the service hours required per patient within 24 hours, according to the professional category and the work shift.Ribeirão Preto, SP, Brazil, 2014

*Quotation from March US$ 1,00 = R$ 2,33

The cost of hours of care per patient spent within 24 hours was US $ 54.24 for nurses and US $ 102.59 for nursing technicians, totaling $ 156.83. Regarding the required hours, the values obtained were US $ 76.73 for nurses and US $ 146.41 for technicians, totaling US $ 223.14. 

The difference in cost value of the nurses' time required as compared with the current is US $ 22.49, which corresponds to an increase of 41.46%. For nursing technicians, the increase is US $ 43.82, corresponding to 42.71%. This means a daily increase of US $ 66.31 (42.28%) per patient.

From the monthly wage composition values of different categories of nurses in the unit, the calculation of the cost of the available and required staffing structure in the ICU is described in Table 4.

**Table 4:**
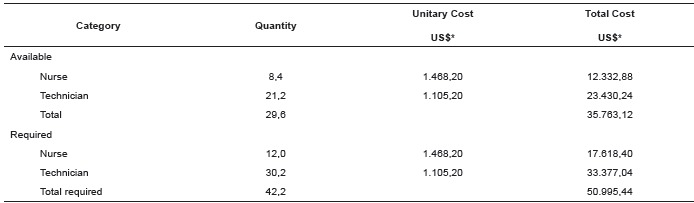
Monthly cost of the average nursing professionals: quantity available and required in the Intensive Care Unit (ICU) Ribeirão Preto, SP, Brazil, 2014

*Quotation from March 2014: USD 1.00= 2.33 R$

## Discussion

The knowledge of the characteristics of hospitalized patients, as well as the evaluation of the demands and care of these patients, enabled us to identify the nursing workload with a NAS daily mean score of 85.6, pointing to a quantitative deficit and the need to adjust the staff structure in 30%. This reality has been investigated and described in other national studies that show a similar picture, of health institutions operating with insufficient nursing staff, particularly in the critical care units^12^ ^-^ ^14^.

It is noteworthy that the minimum standards effective in the country for the different levels of training of nursing professionals show a 1: 2 for nursing technicians and 1:10 for nurses^15^. However, the international literature states a requirement following a gold standard in intensive care with a ratio of 1:1 (nurse / patient). These different professional arrangements are made in specific realities^16^.

Evidence shows the direct relationship between compliance of nursing staff and care and the outcomes related to patient safety and nursing workers safety. These data have been published by professional associations, demonstrating the importance of the right dimension of the professional staff^9^.

A review study that evaluated the association of the nursing framework with the results of the care provided to patients in intensive care units showed that the reduced nurse staffing is associated with the occurrence of adverse events in critically ill patients^17^. 

In this sense, an American study investigated adverse events in critically ill patients and demonstrated that the costs associated with these clinical outcomes are considerable and justify greater investment in prevention strategies^18^.

Despite the assumption that the human resources impact the nursing care management, there is a consensus among nursing managers about the resistance encountered to have the right amount of professional staff in health institutions, mainly due to budgetary reasons^19^.

This reality shows us that the financial support has limited the supply of professionals for healthcare coverage, and this highlights the importance of building evidence to assist nurses in their explanatory efforts regarding the financial impact of a team of professionals that is not consistent with the care needs of the unit.

This strategy becomes the basis of a service management model in the ICU, where knowledge and the application of legal requirements must accompany the guarantee of the kind of care that is provided. The social results presented by the healthcare units is more important than the financial ones, even though this does not preclude the existence of an information system that allows a wide view and management of the costs of the activities and the professionals involved in their performance.

While acknowledging the urgency of studies that address the ICU care costs, few investigations have been completed, especially those involving the nursing staff, because of the difficulty of data quality and non-standard choice and use of methodologies, preventing comparisons of results^20^ ^-^ ^21^. It appears that the approaches traditionally used to estimate costs are based on the calculation of the average cost per patient or patient-day with the annual budget data and indirect costs of ICU, divided by the number of patients, and this sequence assumes the same cost for all patients^22^.

The systematic assessment of personnel costs coming to a fixed value, although it is a simple method for estimating the cost of procedures or of each patient's stipend presents disadvantages, from the budget point of view, as to its application in the ICU, since the standard charging system do not offer a dynamic view of the costs per patient, per admission status, per day of week^23^. 

The national^23^ and international literature^20^ ^)^ showed that the cost of staff is variable as there are patients with different care complexities, and it is possible to demonstrate these oscillations by incorporating the nursing staff workload measurement to the individual staffing cost methodologies. For individual estimates of personnel costs, the authors measure the workload through the *Therapeutic Intervention Scoring System (TISS-28),* finding significant differences in levels of hospital care. In this sense, a research in a Neonatal inpatient unit of a university hospital, which used the NAS as a measure of workload, concluded that the amount necessary for the adequacy of the nursing professional staff would be 30%^7^. 

The NAS covers 80.8% of the nursing activities in contrast to other instruments such as the TISS, comprising 43.3%^6^, and it should be chosen to quantify the nursing activities that have a major impact on the total cost of ICUs and can serve as a monitoring tool for the Unit. It is able to better discriminate the nursing participation in the total cost of the unit, making it possible to identify the differences of individual costs of patients^1^.

Considering the difficulties for health cost control and in particular for intensive care units, as they constitute a substantial financial burden on health systems, there is the need to produce more research to enable better understanding of the cost -benefit ratio in addition to the demand for intensive care and supply of beds, in order to avoid growth while experiencing restrictions in the costs of care for critical patients^24^. 

From this perspective, this research brings a contribution to the managers of nursing services using a detailed methodology in terms of operational steps and feasible to obtain the financial data that is needed. It represents a potential tool that enables its applicability in health institutions of different countries facing the same reality, regarding staffing nurses in critical care units in different arrangements of these teams.

It is important to highlight that among the limitations in such studies, involving a cost analysis of a given professional category in the Brazilian reality, the absence of information on wage rates, due to the lack of a minimum wage for the category of professional nursing, which can lead to differences and outliers on the results, depending on the institution. It is recommended to reproduce this research in other scenarios of intensive care, such as in private hospitals, allowing the construction of their own results, thus enabling mechanisms of *benchmarking* and contributing with additional information on the methodology.

## Conclusion

Using the instrument *Nursing Activities Score* (NAS) in this study, as variable for the staffing dimension of the nursing team, proved relevant as a tool in the cost identification process of nursing care in intensive care, providing support to nurse managers and administrators for planning and budgeting applications. 

The identification of hours spent and required on nursing care by patients in the studied ICU combined to the cost of direct labor, made it possible to meet the financial allocation for adaptation of the quantitative nursing professionals whose monthly financial expense for the institution would amount to US $ 15,232.32, representing an increase of 42.59% in the budget of the unit. From these considerations and the advancement of studies in planning of nursing staff as well as the strategic component that health costs represent, it is deemed necessary to deepen in the study of this topic, expanding the focus of the understanding of institutional management.

## References

[1] Miranda DR, Jegers M (2012). Monitoring costs in the ICU: a search for a pertinent
methodology. Acta Anaesthesiol Scand.

[2] Debergh DP Myny D, Van Herzeele I Van Maele G, Reis Miranda D Colardyn F (2012). Measuring the nursing workload per shift in the ICU. Intensive Care Med.

[3] Coelho FUA, Queijo AF, Andolhe R, Gonçalves LA, Padilha KG (2011). Carga de trabalho de enfermagem em Unidade de Terapia Intensiva de
cardiologia e fatores clínicos associados. Texto Contexto Enferm.

[4] Rossetti AC, Gaidzinski RR (2011). Estimativa do quadro de pessoal de enfermagem em um novo
hospital. Rev. Latino-Am. Enfermagem.

[5] Queijo AF, Padilha KG (2009). Nursing Activities Score (NAS): adaptação transcultural e validação
para a língua portuguesa. Rev Esc Enferm USP.

[6] Miranda DR, Nap R, de Rijk AMA, Schaufeli WMA, Iapichino GMD (2003). Nursing Activities Score (NAS).. Crit. care med.

[7] Ducci AJ, Padilha KG (2008). Nursing Activities Score: estudo comparativo da aplicação
retrospectiva e prospectiva em Unidade de Terapia Intensiva. Acta paul. enferm.

[8] Fugulin FMT, Lima AFC, Castilho V, Bochembuzio L, Costa JÁ, Castro L (2011). Custo da adequação quantitativa de profissionais de enfermagem em
Unidade Neonatal. Rev Esc Enferm USP.

[9] Lima AFC, Castilho V (2015). Mobilização corporal para prevenção de úlceras por pressão: custo
direto com pessoal. Rev Bras Enferm.

[10] Ministério da Fazenda (2015). Banco Central do Brasil.

[11] Fugulin FMT, Lima AFC, Castilho V, Guimarães CP, Carvalho A, Gaidzinsk RR (2015). Quadro de profissionais de enfermagem em unidades médico-cirúrgicas de
hospitais de ensino: composição e custos. Rev Esc Enferm USP.

[12] Wolff LDG, Mazur CS, Wiezbicki C, Barros CB, Quadros VAS. (2007). Dimensionamento de pessoal de enfermagem na unidade semi-intensiva de
um hospital universitário de Curitiba. Cogitare Enferm.

[13] Inoue KC, Matsuda LM (2010). Dimensionamento de pessoal de enfermagem em Unidade de Terapia
Intensiva para adultos. Acta Paul de Enferm.

[14] Girardello DTF, Nicola AL, Fernandes LM (2013). Assistência de enfermagem: horas requeridas para o cuidado do paciente
crítico. Rev Rene.

[15] Ministério da Saúde (BR). Agência Nacional de Vigilância
Sanitária (2012). RDC nº 26, de 11 de maio de 2012. Altera a Resolução RDC nº 07, de 24 de
fevereiro de 2010, que dispõe sobre os requisitos mínimos para funcionamento de
Unidades de Terapia Intensiva e dá outras providências.

[16] Pilcher J, Odele M (2000). Position statement on nurse patient ratio in critical
care. Nurs Stand.

[17] Penoyer DA (2010). Nurse staffing and patient outcomes in critical care: a concise
review. Crit. care med.

[18] Kaushal R, Bates DW, Franz C, Soukup JR, Rothschild JM (2007). Costs of adverse events in intensive care units. Crit. Care Med.

[19] Magalhães AMM, Riboldi CO, Dall´Agnol CM (2009). Planejamento de recursos humanos de enfermagem: desafio para as
lideranças. Rev Bras Enferm.

[20] Moerer O, Plock E, Mgbor U, Schmid A, Schneider H, Wischnewsky MB (2007). A German national prevalence study on the cost of intensive care: an
evaluation from 51 intensive care units. Crit Care.

[21] Tan SS, Bakker J, Hoogendoorn ME, Kapila A, Martin J, Pezzi A (2012). Direct Cost Analysis of Intensive Care Unit Stay in Four European
Countries. Applying a Standardized Costing Methodology.

[22] Pittoni G, Scatto A (2009). Economics and outcome in the intensive care unit. Curr Opin Anaesthesiol.

[23] Telles SCR, Castilho V. (2007). Staff cost in direct nursing care at an intensive care
unit. Rev. Latino-Am. Enfermagem.

[24] Gooch RA, Kahn JM (2014). ICU Bed Supply, Utilization, and Health Care Spending - An Example of
Demand Elasticity. J. am. med. assoc.

